# Predicting ADMET Properties from Molecule SMILE: A Bottom-Up Approach Using Attention-Based Graph Neural Networks

**DOI:** 10.3390/pharmaceutics16060776

**Published:** 2024-06-07

**Authors:** Alessandro De Carlo, Davide Ronchi, Marco Piastra, Elena Maria Tosca, Paolo Magni

**Affiliations:** Dipartimento di Ingegneria Industriale e dell’Informazione, Università degli Studi di Pavia, 27100 Pavia, Italy; alessandro.decarlo01@universitadipavia.it (A.D.C.); davide.ronchi02@universitadipavia.it (D.R.); marco.piastra@unipv.it (M.P.); elenamaria.tosca@unipv.it (E.M.T.)

**Keywords:** model-based drug development, ADMET prediction, graph neural network, attention-based architecture

## Abstract

Understanding the pharmacokinetics, safety and efficacy of candidate drugs is crucial for their success. One key aspect is the characterization of absorption, distribution, metabolism, excretion and toxicity (ADMET) properties, which require early assessment in the drug discovery and development process. This study aims to present an innovative approach for predicting ADMET properties using attention-based graph neural networks (GNNs). The model utilizes a graph-based representation of molecules directly derived from Simplified Molecular Input Line Entry System (SMILE) notation. Information is processed sequentially, from substructures to the whole molecule, employing a bottom-up approach. The developed GNN is tested and compared with existing approaches using six benchmark datasets and by encompassing regression (lipophilicity and aqueous solubility) and classification (CYP2C9, CYP2C19, CYP2D6 and CYP3A4 inhibition) tasks. Results show the effectiveness of our model, which bypasses the computationally expensive retrieval and selection of molecular descriptors. This approach provides a valuable tool for high-throughput screening, facilitating early assessment of ADMET properties and enhancing the likelihood of drug success in the development pipeline.

## 1. Introduction

Drug discovery and development is a difficult, time-consuming, intricate and costly task that is plagued with a considerable amount of doubt as to whether a drug will actually be successful. According to Wouters et al., who studied the data on new therapeutic agents approved by the FDA between 2009 and 2018, the average cost of developing a single new drug is estimated to easily exceed 2 billion dollars [1]. Despite the significant investments in time, resources and money, there is no guarantee that a drug will be approved, and failure can occur during many phases of drug development. Cook et al. [2] comprehensively reviewed the results of AstraZeneca small-molecule drug projects from 2005 to 2010 and found that undesirable absorption, distribution, metabolism, excretion and toxicity (ADMET) properties are a leading cause of failure in the clinical phase of drug development. In response to these findings, there has been a growing trend in the use of in vitro and in vivo ADMET prediction methods [3,4,5]. These methods aim to predict the ADMET properties of a drug before it enters clinical trials, allowing researchers to identify and address potential problems early on in the development process. This can save time and resources as well as increase the chances of a drug being approved.

However, performing complex and expensive ADMET experiments on a large number of compounds is impractical [6,7]. Therefore, multiple in silico strategies have been proposed to predict ADMET properties without the need for in vitro and in vivo experiments [8,9,10]. In silico approaches offer several advantages over experimental methods, including lower costs and the ability to process a large number of compounds in a high-throughput manner [11]. For decades, the development of quantitative structure–activity relationship (QSAR) models has aimed to link chemical information with biological properties and shed light on the interactions between ligands and biological targets. However, it is only with recent advancements in powerful computational techniques that the application of QSAR models has been able to expand and address more complex challenges, such as predicting the ADMET properties of molecules [12]. In addition to the advancement in computational power, another key step has been made with regard to instrumentation and quantification methods that enable large numbers of molecules to be screened, resulting in the generation of large datasets that have been used for artificial intelligence (AI)-based methods.

The application of AI in the ADMET field is rapidly advancing, and a wide variety of models has been developed to predict various properties of drug candidates. These models leverage different AI algorithms, including machine learning (ML) and deep learning (DL) techniques, to analyze large amounts of data and identify promising drug candidates. Some examples of ML algorithms used in ADMET research include random forest [13], support vector machines [14], artificial neural networks [15,16] and k-nearest neighbors (k-NN) [17]. These algorithms are often used to predict important ADMET properties such as solubility, permeability and toxicity, among others. DL algorithms are becoming increasingly popular in ADMET research due to their ability to model intricate connections between molecular attributes and these essential drug properties. Examples of DL algorithms used in ADMET research include recurrent neural networks (RNNs) [18] and generative adversarial networks (GANs) [19]. These algorithms can model complex interactions between drugs and biological systems, leading to more accurate predictions.

It is worth noting that the majority of AI algorithms used for predicting ADMET properties rely on molecular descriptors as input [20]. While these descriptors provide valuable information about the chemical and physical properties of drug candidates, they may not capture the full complexity of ADMET processes. Molecular descriptors are mathematical representations of molecular structures and properties such as size, shape and charge [21]. They are widely used as input for machine learning (ML) models, as they can be easily computed from molecular structures and processed by different types of algorithms. The use of molecular descriptors as inputs to AI models for ADMET prediction has several limitations. One limitation is that molecular descriptors provide a simplified representation of the molecular structure and may not capture all relevant features that affect ADMET properties [22]. Another limitation is that molecular descriptors are derived from specific algorithms and calculations, which can differ among studies and may not be consistent across different datasets [23]. This can lead to discrepancies in ADMET predictions and make it difficult to compare results across sources. Recently, a deep learning (DL) approach based on graph neural networks (GNNs) has been proposed to predict molecular thermophysical properties [24] and has achieved very interesting performance. This framework leverages the flexibility of graph theory to represent structural relationships among the atoms in a molecule and, thus, to circumvent the use of molecular descriptors. Other preliminary analyses showed that this methodology can reach good performance when predicting some drug ADMET properties [25,26].

The aim of this paper is to develop and evaluate an attention-based graph neural network (GNN) approach for predicting the ADMET properties of molecules. The proposed approach leverages only the molecular structure information that can be obtained from the Simplified Molecular Input Line Entry System (SMILES) [27] and does not require the calculation of molecular descriptors. This makes it computationally efficient and enables the prediction of ADMET properties for large compound libraries in a time-effective manner. The newly introduced model examines both the entire molecular structure and its substructures with an attention-based approach; thus, both global and local features are used to infer drug ADMET properties. The architecture is evaluated with five-fold cross validation (CV) on large (i.e., more than 4200 compounds) publicly available regression/classification datasets of ADMET properties on which similar DL approaches have been tested. Furthermore, to provide a more comprehensive assessment of the novel features (i.e., the graph attention mechanism for both the entire molecule and its substructures), an ablation study of the newly proposed GNN architecture and the standard test procedure of the Therapeutics Data Commons (TDC) platform [28] are performed.

## 2. Materials and Methods

### 2.1. Molecular Graph Representation

The pillar of the work presented here is that each molecular structure can be represented as a graph [29]. Formally, a graph, G=(V,E), is a data structure defined by a set of nodes V linked by a set of edges E representing connections between nodes. In molecular graphs, each node $v_{i}\in V$ represents an atom of the molecule, and each edge $e_{l}\in E$ represents a bond between atoms. Within molecular graphs, edges are usually associated with bidirectional characteristics. As a result, the graph under consideration is an *undirected graph*, where the connections between nodes do not have specific directions.

The theoretical definition of a graph can be translated into a computer-processable representation by leveraging linear algebra and matrices [29]. The connections between atoms in a molecule are typically represented by an adjacency matrix $A\in R^{N\times N}$, where N=|V| is the number of atoms in the molecule. Atomic bonds are defined in A by setting $a_{ij}=1$ if atoms/nodes $v_{i}$ and $v_{j}$ are linked; otherwise, the value is set to 0 [29]. Consequently, if the graph is undirected, A is a symmetric matrix [30]. In the adopted graph representation, each node/atom is assumed to be connected to itself [30]. Therefore, elements along the diagonal of A, $a_{ii}$ are set to 1.

**Figure 1:**
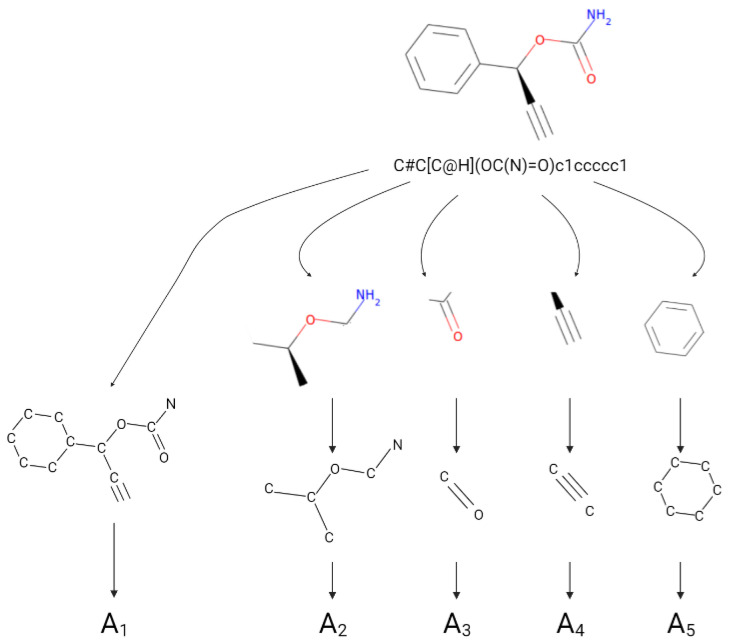
Example of how adjacency matrices are extracted from molecular SMILE. For each type of bond (i.e., single, double, triple or aromatic), a specific adjacency matrix is derived in order to focus on molecular substructures.

As discussed in Section 2.2, the implemented deep learning model focuses on both the whole molecule and its substructures. As illustrated in Figure 1, it is necessary to consider a set of adjacency matrices for each molecule. For evaluating the entire molecular composition, a first adjacency matrix $A_{1}$ is defined by considering bonds of all kinds. In addition, four other adjacency matrices, $A_{2}$, $A_{3}$, $A_{4}$ and $A_{5}$, are derived for each molecule in order to focus on substructures characterized only by single, double, triple and aromatic bonds between atoms, respectively. In the proposed implementation, the dimensions of all the adjacency matrices are taken to be N×N, independent of the number of atoms that appear in the specific substructure.

Within the framework of molecular graphs, nodes are not only described by their interconnections but also by considering their intrinsic characteristics. Therefore, each node $v_{i}\in V$ is described by a feature vector, $h\in R^{D}$, which contains information about the specific atom of the molecule (e.g., type, formal charge, etc.) [29]. All the feature vectors can be stored within a node feature matrix, $H\in R^{N\times D}$, whose rows, $h_{i}$ with i=1,…,N, are the features associated with each atom in the molecule [29,30]. Table 1 summarizes the atomic features considered in our work. It is important to underline that all these atomic characteristics can be derived from the chemical composition of a compound without using the predictions of other models. Each feature is described with a one-hot encoded vector: the concatenation of these vectorial representations defined the final feature vector associated with each atom.

In our approach, the molecular graph representation described above is obtained from SMILES through a specific pre-processing pipeline. A detailed description of the entire process is reported in Supplementary Materials S2.

### 2.2. Graph Neural Networks (GNNs)

**Figure 2:**
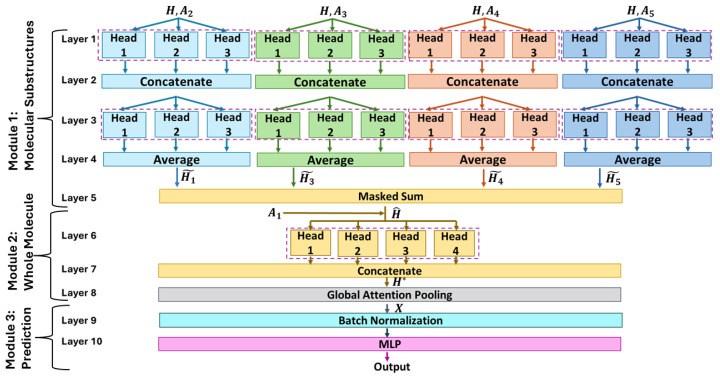
Schematic representation of the GNN adopted. The architecture is organized as a stack of three main modules, each with a specific function.

A graph neural network (GNN) is a deep learning framework that processes graph input data [31,32] for both classification and regression tasks, which can be performed at different levels: on the entire graph, on single nodes, or on edges [30]. According to the molecular graph representation described in Section 2.1, the proposed application of GNN falls under graph-level classification and regression tasks. Figure 2 describes the GNN architecture developed in this work.

The GNN architecture can be subdivided into three modules:

- **Module 1: Layers 1–5** focus on molecular substructures. The first four layers are characterized by four independent and parallel branches, each considering an adjacency matrix $A_{k}$, with k=2,3,4,5, that represents a particular substructure of the molecule as defined in Section 2.1. Each branch *k* uses its adjacency structure, $A_{k}$, to remap into a different feature space the input node feature matrix $H\in R^{N\times D}$, which was built by concatenating the one-hot representations of the atomic characteristics in Table 1. Thus, after Layer 4, four new node feature matrices, ${\tilde{H}}_{k}\in R^{N\times F}$, are obtained. Each ${\tilde{H}}_{k}$ provides a latent representation of the original feature matrix by considering a specific molecular substructure represented through the adjacency matrix $A_{k}$. The projection of H into ${\tilde{H}}_{k}$ through $A_{k}$ is performed by each branch combining two multi-head attention layers (MHALs), located in both Layer 1 and 3, with the operations of concatenation (Layer 2) and averaging (Layer 3). A more detailed description of these layers is reported in Section 2.2.1. The outputs of all four branches are then combined into Layer 5 with a masked sum (Section 2.2.2) to obtain a new node feature matrix $\hat{H}\in R^{N\times F}$ that merges the information ${\tilde{H}}_{k}$ coming from the different substructures.
- **Module 2: Layers 6–8** consider the whole molecular structure. The inputs of this module are $\hat{H}$ and $A_{1}$, the latter being the adjacency matrix built considering all bond types simultaneously. $\hat{H}$ and $A_{1}$ are fed into another MHAL (Layer 6) whose outputs are then concatenated (Layer 7), leading to new node feature matrix $H^{*}\in R^{N\times Q}$. Finally, Layer 8 projects into a *P-dimensional* space the node feature matrix $H^{*}$ and then squeezes it into a vector $X\in R^{P}$ representing the graph-level features.
- **Module 3: Layer 9–Output** leverage the layers of fully connected neural networks. In this module, X is fed into a batch normalization layer [33] and then to a multi-layer perceptron (MLP) [34] that yields the final prediction.

From this modular description of the GNN architecture, it follows that the model uses a *bottom-up* approach for inferring molecular characteristics. Indeed, Module 1 focuses on the internal structures (i.e., subgraphs) for extracting a new representation of the node feature matrix $\hat{H}$. Then, in Module 2, the whole molecule (i.e., full graph) is accounted for, achieving a graph-level representation with vector X. Finally, Module 3 is used to predict the molecular property of interest.

#### 2.2.1. Multi-Head Attention Layer

The multi-head attention layer (MHAL) was first introduced for deep neural network architectures to improve performance in sequence-based tasks related to the field of computational linguistics, such as machine translation [35] and language modeling [36]. In particular, this mechanism allows the net to understand which subset of elements in a sequence is more important for the final prediction. The introduction of the Transformer model [36] made the attention mechanism a widely adopted solution for several tasks [37]. The strength of the Transformer model is the presence of multiple and independent attention units (i.e., heads), each of which focuses on different aspects of the same input. Analogous to [38], here, the multi-head attention mechanism is combined with the GNN framework to obtain a new representation for each node/atom using different attention scores based on the contributions coming from the neighborhood of the node/atom. This approach represents an extension of the classical graph convolutional (GC) layers, for which a description is reported in Supplementary Materials S3.

In our approach, each head takes as input a generic graph, G=(V,E), which is represented with an adjacency matrix, $A\in R^{N\times N}$, describing the node connections and a node feature matrix H. The *j-th* node, $v_{j}$, is characterized by a set of neighbors *U*, also including $v_{j}$ itself, and by a feature vector $h_{j}\in R^{D}$ (i.e., the *j-th* row of H). This head is characterized by a trainable matrix of weights, $W\in R^{D\times F}$, which is used to perform an initial linear transformation of H. This procedure leads to a new node feature matrix belonging to an *F-dimensional* feature space, with *F* representing a hyperparameter.

(1) Z=H·W

Consequently, $v_{j}$ will be characterized by a new feature vector, $z_{j}\in R^{F}$. Then, the attention coefficients, $e_{jl}$, that quantify the importance given by $v_{j}$ to the features of each $v_{l}\in U$ are computed as:

(2) $$e_{jl}=g\left(\left(z_{j}⊕z_{l}\right)\cdot a^{T}\right).$$

This operation is performed by concatenating (⊕ symbol) the transformed features of $v_{j}$ and $v_{l}$ and then by performing the scalar product with vector $a\in R^{2F}$. In particular, a is a trainable parameter vector, while *g* is a non-linear function (i.e., activation function). In the implemented model, *g* was set to be a LeakyReLU function [39] with slope α=0.2, as in [38].

Attention coefficients are then normalized as reported in Equation (3).

(3) $$\lambda_{jl}=\frac{\exp(e_{jl})}{\sum_{v_{l}\in U}\exp\left(e_{jl}\right)}$$

Values $\lambda_{jl}$ are subsequently used in Equation (4) to compute the new feature vector, ${\tilde{h}}_{j}\in R^{F}$, for the node $v_{j}$ and, in general, the new feature matrix $\tilde{H}\in R^{N\times D}$.

(4) $${\tilde{h}}_{j}=\phi \left(\sum_{v_{l}\in U}\lambda_{jl}z_{l}\right)$$

More specifically, ${\tilde{h}}_{j}$ is obtained by applying a nonlinear function, ϕ (also in this case a LeakyReLU with α=0.2), to the weighted sum in the *F-dimensional* space between the features of $v_{j}$ and the ones of its neighbors. Therefore, $\lambda_{jl}$ gives an attention-based weight to the neighborhood of $v_{j}$ and to $v_{j}$ itself, since the node’s self-attention was previously computed.

Equations (1)–(4) describe a single attention head; these are further combined to create *K* attention heads (the *K* hyperparameter). While the input is shared, each head, independently of the others, processes the same input and returns a new node feature matrix ${\tilde{H}}^{K}$. The final output of the MHAL is the average of the *K* matrices (Layer 4 of the model in Figure 2) or a concatenation (Layers 2 and 8 in Figure 2). While the first solution maintains an *F-dimensional* feature space accordingly to the output of each head, the latter expands it to a K· *F-dimensional* representation.

#### 2.2.2. Masked Sum Layer

This layer was introduced in the architecture (Layer 5 of Figure 2) to integrate the contributions of the pre-processing coming from the four branches k=2,3,4,5, which each focus on a molecular substructure. It takes as input the set of the adjacency matrices $A_{k}\in R^{N\times N}$ and those of the feature matrices ${\tilde{H}}_{k}\in R^{N\times F}$. Given the set of nodes V, with |V|=N, for each $v_{j}\in V$, a new feature vector ${\hat{h}}_{j}\in R^{F}$ is computed with Equation (5).

(5) $${\hat{h}}_{j}\;=\sum_{k=2}^{5}{\tilde{h}}_{k,j}\cdot a_{k,jj}$$

In particular, by multiplying ${\tilde{h}}_{k,j}$ with the *j*-th element on the diagonal of $A_{k}$, it is possible to compute the features of node $v_{j}$ by considering the contributions of the substructures to which it belongs.

#### 2.2.3. Global Attention Pooling Layer

This layer was introduced in the architecture (Layer 7 of Figure 2) to obtain a single feature vector $X\in R^{P}$ summarizing all graph features. As reported in [40], this layer takes as input a feature matrix $H^{*}$. Following a linear transformation mapping $H^{*}$ from $R^{N\times Q}$ to $R^{N\times P}$, an attention-based sum of all the *N* feature vectors $h_{j}^{*}$ is executed (Equation (6)).

(6) $$X=\sum_{j=1}^{N}\left(\sigma \left(h_{j}^{*}W_{1}+b_{1}\right)⊙\left(h_{j}^{*}W_{2}+b_{2}\right)\right)$$

In particular, $W_{1}$ and $W_{2}$ are two $R^{Q\times P}$ matrices of learnable weights, $b_{1}$ and $b_{2}$ are two $R^{P}$ vectors of other learnable parameters, and σ represents the sigmoid activation function.

### 2.3. Benchmark Datasets

This work utilizes six public datasets, consisting of two regression datasets and four binary classification datasets, covering various ADMET properties. The two regression datasets are the Lipophilicity AZ dataset and the AqSolDB dataset, which contain information on lipophilicity and aqueous solubility. Lipophilicity, expressed in terms of LogD, significantly impacts drug solubility and permeability, thereby affecting its potency and selectivity. In the early stages of drug development, several candidate compounds are characterized by high lipophilicity. However, such high lipophilicity often results in rapid metabolism, leading to poor solubility and diminished absorption. Aqueous solubility (LogS) evaluates the drug’s ability to dissolve in water, impacting mainly its absorption kinetics and bioavailability. Compounds with low aqueous solubility may exhibit slower absorption rates, potentially leading to inadequate therapeutic levels in the bloodstream and reduced efficacy. This limitation is particularly impactful as about 70% of newly developed medications demonstrate poor solubility [41].

Regarding the classification datasets, all of them concern the activity of different cytochromes (CYP P450). This class of enzymes plays a key role in pharmacogenetics and frequently showcases genetic variations that directly impact drug activity. This genetic diversity influences both the pharmacokinetic and pharmacodynamic responses of individuals to medications, affecting both therapeutic outcomes and adverse reactions [42]. There are 57 active CYPs in the human genome, which are denominated using a sequence of a digit, a letter, and a number that represent the gene family, the subfamily, and the gene identifier, respectively. Among them, CYP P450 2C9 plays a significant part in oxidizing both xenobiotic and endogenous compounds. Meanwhile, the CYP2C19 gene directs the production of an enzyme crucial for endoplasmic reticulum function by facilitating protein processing and transport. CYP2D6 is primarily active in the liver but is also prominently present in key areas of the central nervous system such as the substantia nigra. Lastly, CYP3A4, predominantly located in the liver and intestine, serves as a vital enzyme responsible for oxidizing various foreign organic molecules, including toxins and drugs, aiding in their elimination from the body [42].

Statistical information of these datasets is shown in Table 2.

### 2.4. Evaluation of GNN Framework

**Figure 3:**
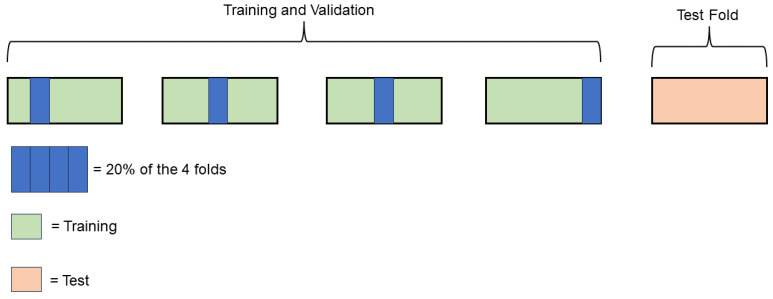
Schematic representation of the implemented five-fold cross validation. At each step, one fold (orange) is used as an external test set; the remaining four are used for training and validation. And 20% of the four folds are used as validation data.

The architecture described in Section 2.2 was tested on both the classification and regression tasks that are described in Section 2.3. In order to provide a robust evaluation of the GNN framework, a five-fold cross validation (5-FCV) approach was conducted on the six tasks. As schematized in Figure 3, at each step of the 5-FCV, the 20% of the data excluded from the test fold is randomly used as a validation set to avoid overfitting during the training [43] phase and to maximize the model’s generalizability. The remaining 80% constitutes the training set. In particular, at the end of each training iteration (i.e., epoch), the model is evaluated on the validation set by using a specific metric. At the end of the training, the final model is the one with the best score on the validation set. The metrics adopted for the regression and classification tasks are reported in Section 2.6.

Training, test and validation sets were defined in a stratified manner for classification tasks due to the unbalanced distributions of the examples (Table 2).

### 2.5. Custom Training Loss Functions

Training a neural network consists of solving an optimization problem in which a set of optimal parameters θ is found by minimizing a cost function (i.e., loss function) $f(y,\hat{y},\theta )$. More specifically, *f* is a function of the model parameters θ, the real value of the target variable *y* and its prediction $\hat{y}$. The definition of *f* depends on the task for which the model is applied (i.e., regression or classification). In particular, given the datasets presented in Section 2.3, we used the root mean squared error (RMSE, Equation (7)) and the binary cross-entropy (BCE, Equation (8)).

(7) $$RMSE:=\sqrt{\frac{\sum_{i=1}^{N}{\left(y_{i}-\hat{y_{i}}\right)}^{2}}{N}}$$

(8) $$BCE:=-\frac{1}{N}\sum_{i=1}^{N}y_{i}\cdot log(p\left(x_{i}\right)+\left(1-y_{i}\right)\cdot log\left(1-p\left(x_{i}\right)\right)$$

In the classification task, examples are labeled with 0 or 1, and the term $p(x_{i})$ in Equation (8) represents the probability that example $x_{i}$ belongs to class 1. As detailed in Section 2.5.1 and Section 2.5.2, the loss functions in Equations (7) and (8) were modified by introducing a weighting strategy that addresses skewed or unbalanced distributions.

#### 2.5.1. Weighted RMSE

**Figure 4:**
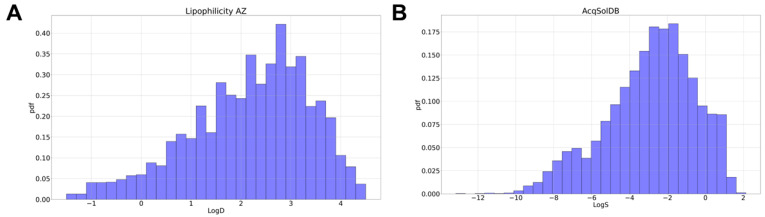
Distributions of regression variables in two benchmark datasets. Histograms of Lipophilicity AZ panel (**A**) and AqSolDB panel (**B**) data.

Training a regression model on a skewed distribution of the target variable (i.e., LogD and LogS, as illustrated in Figure 4) can lead to a biased model that accurately predicts only common cases (i.e., those having a higher probability density) [44,45]. This issue can be addressed by introducing a loss function (in this case, the RMSE) and a weight $w_{i}$ for the *i*-th example according to the probability density of its target value $p(y_{i})$ (Equation (9)) [46].

(9) $$WRMSE:=\sqrt{\frac{\sum_{i=1}^{N}w_{i}{\left(y_{i}-\hat{y_{i}}\right)}^{2}}{N}}$$

In particular, $w_{i}$ is defined as $\propto 1/p(y_{i})$ according to the algorithm proposed in [46]. Given the set of target values, $Y=\{y_{1},y_{2},\ldots ,y_{N}\}$, it is possible to estimate $p(y_{i})$ with a kernel density technique [46,47,48]. Then, $p(y_{i})$ is normalized between 0 and 1 by applying Equation (10).

(10) $$p^{\prime }\left(y_{i}\right)=\frac{p\left(y_{i}\right)-min\left(p\left(Y\right)\right)}{max(p(Y))-min(p(Y))}$$

The weight $w_{i}$ can be computed for each $y_{i}$ with Equation (11), which depends on a design parameter α∈[0,1].

(11) $$w_{i}=1-\alpha p^{\prime }\left(y_{i}\right)$$

**Figure 5:**
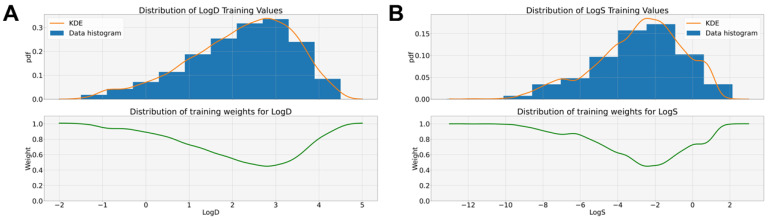
Example of the weighting strategy adopted for both regression tasks. Panel (**A**) shows the weights introduced for training the GNN on LogD prediction. Panel (**B**) focuses on LogS. For both tasks, α was set to 0.55.

Examples of this weighting strategy are reported in Figure 5 for regression tasks for both LogD and LogS. Further implementation details on this weighting strategy are reported in Supplementary Materials S2.

#### 2.5.2. Weighted Binary Cross Entropy

One of the potential pitfalls of machine learning (ML) methods is highly imbalanced datasets. Indeed, these techniques often do not perform well in classifying minority classes, which could be very relevant for the task at hand [49,50]. Classification tasks reported in Section 2.3 fall into such an imbalanced condition since the target class, i.e., the presence of an inhibitory action by a compound on CYP enzymes (encoded with 1), is underrepresented in the dataset (Table 2). Thus, the BCE loss (Equation (8)) was weighted in order to give a higher importance to the examples of the minority class. To this end, for each class c∈C={0,1}, a weight $w_{c}$ was assigned by considering the log ratio between the size of the majority class $N_{c}^{*}=\max_{c\in C}N_{h}$ and that of *c* (Equation (12)) [51].

(12) $$w_{c}=ln\left(\frac{N_{c}^{*}}{N_{c}}\right)+1$$

Therefore, the parameters of the GNN were trained by minimizing the weighted BCE (WBCE) loss function in Equation (13), with $w_{i}\in \left\{w_{0},w_{1}\right\}$ depending on the class c∈C of $y_{i}$.

(13) $$WBCE:=-\frac{1}{N}\sum_{i=1}^{N}w_{i}\cdot \left(y_{i}\cdot log(p\left(x_{i}\right)+\left(1-y_{i}\right)\cdot log\left(1-p\left(x_{i}\right)\right)\right)$$

### 2.6. Validation Set Metrics

The validation set has a crucial role during the training stage to avoid overfitting [43]. At the end of each training epoch, in fact, the model is evaluated on the validation set to detect, as early as possible, the worsening of loss values. Then, at the end of training, the best model parameters are those that achieve the best score on the validation set.

RMSE (Equation (7)) was used as a validation metric for regression tasks (Table 2). This function was preferred to MAE (Equation (14)) as it gives a higher weight to larger model errors.

(14) $$MAE:=\frac{\sum_{i=1}^{N}\left|y_{i}-\hat{y_{i}}\right|}{N}$$

However, as will be discussed in the Results section, MAE was also used to assess the performances of the model on the test set in order to allow comparisons with the other approaches in the literature (Table 3 and Table 4). The goal of the classification tasks presented in Section 2.3 is to accurately detect those molecules inhibiting CYP enzimes (labeled as ‘Positive’ or 1). However, such molecules represent the minority class in the available datasets (Table 2). These aspects lead us to consider the area under the precision–recall curve (AUPRC) as a validation metric rather than the area under the receiving–operating characteristic curve (AUROC) [52,53].

(15) $$Precision:=\frac{\mathrm{True}\;\mathrm{Positives}}{\mathrm{True}\;\mathrm{Positives}+\mathrm{False}\;\mathrm{Positives}}$$

(16) $$Recall:=\frac{\mathrm{True}\;\mathrm{Positives}}{\mathrm{True}\;\mathrm{Positives}+\mathrm{False}\;\mathrm{Negatives}}$$

In particular, the precision–recall (PR) curve focuses on the trade-off between the values attained by each of the two metrics (Equations (15) and (16)) by considering different decision thresholds (i.e., probability threshold for assigning an example to a given class) [54]. Unlike the ROC curve, the PR curve is not influenced by the true negatives, and this is an advantage in the presence of unbalanced datasets. Therefore, the AUPRC represents an evaluation metric targeted to how the model performs on positive cases [54]. Analogously to the AUROC, there is a baseline value for the AUPRC that is the proportion of positive examples in the dataset (i.e., a naive classifier assigning the positive class to all the examples) [54,55].

**Table 3:** Summary of evaluation strategies for references.

Reference	Model	Metrics	Evaluation Strategy
Lipophilicity			
Zhang et al. [56]	BERT transformer adapted to molecular graph structures (MG-BERT)	R2	The model was trained 10 times using random dataset splits, and the final performance was reported as the average with standard deviation.
Wang et al. [57]	Convolutional GNN integrated with feed-forward neural networks (FNNs) processing molecular fingerprints	MAE	Holdout (70%:30%)
Peng et al. [26]	Convolutional GNN based on graph isomorphism [58]	RMSE	5-fold CV on 85% of samples, with the remaining used as an external test set. Each comparison was conducted 20 times, and the final result was the average.
Tang et al. [59]	Graph-based encoder integrated with FNN	RMSE	10-fold CV (80%:10%:10%). All experiments were repeated three times with different random seeds.
Li et al. [60]	Adaptation of LSTM-based model originally developed for natural language processing tasks	RMSE	All the models were evaluated on the test sets using 10 randomly seeded 80:10:10 data splits.
AcqSol			
Xiong et al. [61]	Graph attention neural network processing the entire molecular structure	MAE	TDC-style.
Francoeur et al. [62]	Molecular attention transformer presented in [63]	RMSE	3-fold clustered cross-validation split of the data
Yang et al. [64]	Graph neural networks	MAE	TDC-style.
Venkatraman et al. [65]	Random forests using molecular fingerprints to represent compounds and SMOTE data augmentation	RMSE	Training–test (80/20). On the training test, 5-fold CV to identify the best performing model. Each comparison was run 3 times, and its final experiment result was the average.
CYP			
Plonka et al. [66]	Random forest and molecular fingerprints to represent compounds	AUROC	10-fold CV on 80% of data and data augmentation. 20% of data used as test set.
Xiang et al. [67]	FNN processing molecular fingerprint descriptors of a compound.	AUROC	Holdout with different datasets.

**Table 4:** Comparative performances of the proposed and the literature models on the ADMET properties datasets.

Metric	Reference	Median	Standard Deviation
Lipophilicity			
MAE	This work	0.422	0.019
	Wang et al. [57]	0.440	-
RMSE	This work	0.576	0.031
	Wang et al. [57]	0.738	-
	Peng et al. [26]	0.586	0.015
	Tang et al. [59]	0.571	0.032
	Li et al. [60]	0.625	0.032
R2	This work	0.774	0.031
	Zhang et al. [56]	0.765	0.026
	Wang et al. [57]	0.766	-
AqSol			
MAE	This work	0.749	0.020
	Xiong et al. [61]	0.776	0.008
	Yang et al. [64]	0.762	0.020
	Venkatraman et al. [65]	0.780	-
RMSE	This work	1.14	0.050
	Francoeur et al. [62]	1.459	-
	Venkatraman et al. [65]	1.12	-
R2	This work	0.767	0.023
	Venkatraman et al. [65]	0.78	-
CYP P450 2C9			
AUROC	This work	0.894	0.009
	Plonka et al. [66]	0.91	-
	Xiang et al. [67]	0.799	-
AUPRC	This work		0.01
CYP P450 2C19			
AUROC	This work	0.882	0.006
	Plonka et al. [66]	0.89	-
	Xiang et al. [67]	0.832	-
AUPRC	This work	0.859	0.008
CYP P450 2D6			
AUROC	This work	0.862	0.008
	Plonka et al. [66]	0.92	-
	Xiang et al. [67]	0.878	-
AUPRC	This work	0.676	0.014
CYP P450 3A4			
AUROC	This work	0.887	0.011
	Plonka et al. [66]	0.92	-
	Xiang et al. [67]	0.929	-
AUPRC	This work	0.842	0.014

### 2.7. Benchmarking Methods

Evaluation of AI models is not a straightforward task because of the wide range of different methods used in different studies. Examples of these variations include the use of additional proprietary data and the adoption of different validation techniques.

Amid these challenges, another aspect contributing to variability is the selection of evaluation metrics. Commonly employed metrics like RMSE, MAE and R-squared (R2) values are frequently adopted to gauge the performance of regression models. Yet using distinct metrics can yield differing outcomes and interpretations of model effectiveness. This divergence complicates direct comparisons among diverse AI models devised for predicting ADMET properties.

To tackle this intricacy, we meticulously reviewed the existing literature to identify prior studies that evaluated AI models on similar datasets and under analogous evaluation methodologies whenever possible. For each specific prediction task, the most promising results were gleaned from the literature (Table 4) and reported with the optimized metrics and the evaluation approach employed in Table 3.

Furthermore, a comprehensive juxtaposition of the performances of the model proposed here against the results documented in the TDC (Therapeutics Data Commons) database was conducted, as expounded upon in Supplementary Materials S1.

### 2.8. Implementation and Code Availability

The GNN framework presented here was developed using Python version 3.7. In particular, the layers of the network were implemented with the TensorFlow 2.4 library (https://www.tensorflow.org, accesssed on 29 May 2024). Scikit-learn utilities were leveraged for the 5-FCV evaluation, and *rdkit* and *networkx* were adopted to obtain the molecular graph representation described in Section 2.1. All codes are fully available on the GitHub repository at the following link: https://github.com/AlessandroDeCarlo27/GNN (accesssed on 29 May 2024).

## 3. Results

The results are summarized in Table 4, while details about the robustness of the inferences, assessed via the five-fold method, are reported in Appendix A. The GNN hyperparameters used for the different tasks are reported in Supplementary Materials S2.

Due to the previously discussed differences in optimized metrics and evaluation methodologies, conducting a systematic comparison of the results is indeed a challenging endeavor.

However, some points can be discussed. Considering the lipophilicity task, the proposed algorithm showed better performances compared to the majority of the benchmarked methodologies. Only the study by Tang et al. [59] exhibits a minor advantage over our results. A more relevant comparison can be done with the work of Wang et al. [57]. Differently from the algorithm proposed here that considered the RMSE as the primary metric of interest, the study conducted by Wang et al. placed its primary emphasis on minimizing the MAE. Then, as expected, the proposed algorithm showed better performance than Wang’s in terms of RMSE (0.576 vs. 0.738). Conversely, when turning attention to MAE, the values are quite close. This favorable outcome can be due the weight we included into the RMSE metric in the loss function.

Considering the AcqSol task, the obtained results are in agreement with the state-of-the-art. When comparing our algorithm with the two available methods that focus on minimizing the MAE, it becomes evident that our algorithm’s performance is marginally better than that of the top-ranking algorithm [61]. Again, it is worth noting that the MAE is not the pivotal metric chosen in this work for optimizing the regression tasks. From the comparison with the work by Venkatraman et al. [65], which specifically aimed to minimize the RMSE, a parallel observation akin to that in the lipophilicity task comes to the fore. Upon comparing the RMSE, the focal metric of interest for both algorithms, it becomes apparent that the algorithm from the literature yields a marginal improvement (Δ<2%) over our own. In contrast, a closer examination of the MAE values demonstrates that Venkatraman’s algorithm yields slightly less favorable results in comparison to ours, underlining once again the generalizability of our method across evaluation metrics.

Moving to the analysis of the classification tasks, a similar observation can be extended across all of the CYP activity tasks. In this context, the performance of our algorithm demonstrates minimal lag behind the leading approach documented in the literature, particularly in terms of AUROC. However, this difference can be attributed to the optimized metric. In this regard, our preference leans towards utilizing the AUPRC as our primary evaluation metric, with AUROC being a consequential derivative metric as discussed in Section 2.6. The distinction between AUPRC and AUROC becomes more pronounced in cases of imbalanced datasets, a characteristic that our results effectively reflect (i.e., CYP P450 2D6). By prioritizing the AUPRC, we align our methodology with the idea that, in this case, maximizing the number of true positives is more important than maximizing the number of true negatives.

### Ablation Study

To demonstrate the validity of the proposed model, an ablation study was conducted on the presented GNN architecture (Figure 2). The main purpose of this study was a comprehensive analysis of the main specific features that are included in the proposed GNN architecture. This study was performed by comparing the performances of the *Complete* GNN architecture against modified variants in which a few crucial features were simplified.

**Figure 6:**
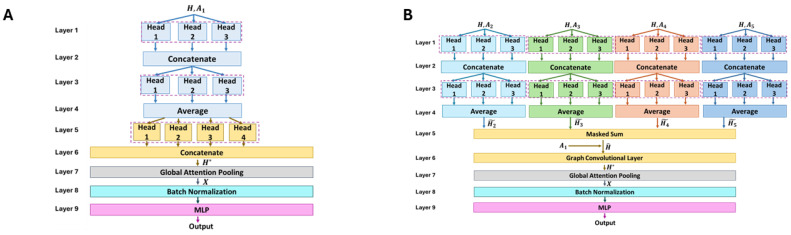
Models used in the ablation study to benchmark the implemented GNN architecture. Panel (**A**) illustrates the ‘Whole Molecule’ GNN, which does not consider molecular substructures. Panel (**B**) represents the ‘Convolutional’ GNN, in which the attention mechanism for the entire molecule is replaced by a graph convolutional (GC) layer.

In particular, two variants of the Complete GNN were considered, as illustrated in Figure 6. The *Whole Molecule* variant (Figure 6, Panel A) was introduced to assess the possible advantages of explicit processing of molecular substructures. This variant, in fact, focuses on the entire molecule structure, as it takes as input the complete adjacency matrix, $A_{1}$. In addition, the *Convolutional* GNN variant (Figure 6, Panel B) was introduced to evaluate the role of the attention mechanisms that act on the complex of substructures in the Complete GNN version. The Convolutional GNN differs from the Complete architecture (Figure 2) in layer 6, in which a graph convolutional (GC) layer replaces the four-head graph attention layer. As detailed in Supplementary Materials S3, the GC layer is the simplest type of layer processing a graph input, and it is characterized by a lower number of parameters than a multi-head attention layer [30].

**Figure 7:**
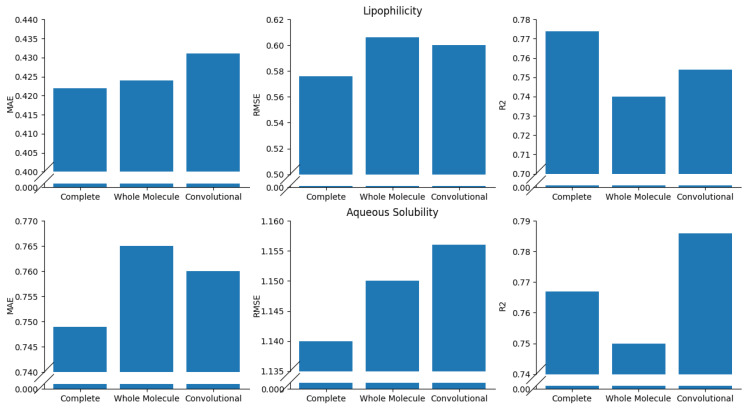
Results of the ablation study on the regression tasks.

**Figure 8:**
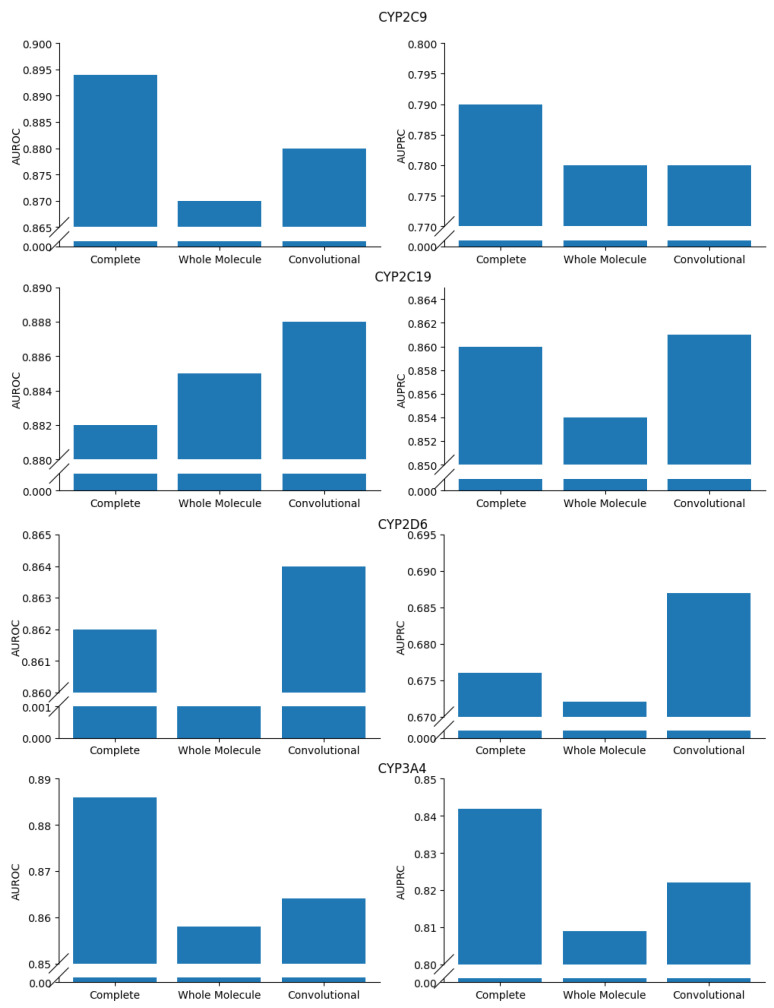
Results of the ablation study on the classification tasks.

Both variants, the Whole Molecule GNN and the Convolutional GNN, were tested with the five-fold CV approach described in Section 2.4 on the same regression/classification tasks on which the Complete architecture was challenged. Further details on the hyperparameters used for the Whole Molecule and Convolutional GNNs are reported in Tables S3.1 and S3.2 of Supplementary Materials S3. Figure 7 and Figure 8 summarize the results of the ablation study. In particular, the Complete GNN architecture always achieved better RMSE/MAE and AUPRC values than the Whole Molecule model on regression and classification tasks, respectively. This result confirms that differentiating the analysis of the molecular substructures in the GNN improves the prediction of ADMET properties. The Complete model outperformed the Convolutional variant in almost all tasks, thus confirming the relevance of attention mechanisms applied to the complexes of molecular substructures. Nonetheless, on two classification problems (i.e., inhibition of CYP2C19 and CYP2D6), the Convolutional variant achieved better AUPRC values than the Complete GNN. This seems to suggest that, at least for some cases, further tuning of hyperparameters in the attention layer (e.g., number of attention heads and/or the dimension of the output latent space) may be required.

## 4. Discussion

The realm of ADMET prediction has witnessed transformative advancements with the advent of artificial intelligence models. These models hold the promise of revolutionizing drug discovery and development by enabling the precise characterization of vital drug properties. However, the journey towards harnessing the full potential of AI-driven ADMET prediction is interleaved by challenges in evaluating the performance of these models. The need for standardized evaluation procedures renders the identification of superior models a difficult task. Discerning whether performance differences stem from genuine model enhancements or disparate evaluation techniques becomes a critical consideration. The quest for robust model comparison necessitates the alignment of evaluation methodologies across studies.

In the study presented here, these challenges were addressed. We identified studies with similar datasets and evaluation methods through an exhaustive literature review. Most top-performing works used graph representations of molecules for regression tasks. Conversely, models for CYP classification primarily relied on processing fingerprint descriptors.

Across tasks like molecular lipophilicity and aqueous solubility, our method consistently outperforms or performs very closely to benchmarked approaches. Moreover, when compared with state-of-the-art algorithms, our model consistently achieves strong results across diverse evaluation metrics. In classification tasks, our strategy of prioritizing AUPRC underscores our dedication to maximizing true positives: a critical aspect for datasets with imbalances. Nonetheless, it is noteworthy that our commitment to AUPRC does not hinder our competitiveness, as we continue to contend with top algorithms even in terms of AUROC.

Furthermore, we conducted an ablation study with two alternative GNN variants to evaluate the design choices made for the Complete GNN architecture. The first variant processed the whole molecule directly, while the second replaced the attention layer with a simpler graph convolutional layer. This analysis confirmed the importance of substructure processing, as the proposed model consistently outperformed the model without it. Additionally, the Complete GNN model also achieved generally better results than the variant using graph convolution, particularly for regression tasks. Nevertheless, our results suggest that for some functions, particularly CYP2C19 and CYP2D6 inhibition prediction, optimizing the attention layer could improve performance. As a final validation procedure, we extended our comparison to the Therapeutics Data Commons. TDC is a platform for systematically accessing and evaluating machine learning across the entire range of therapeutics. TDC provides AI-ready datasets and learning tasks together with an ecosystem of tools, libraries, leaderboards and community resources. Since TDC includes works presented in preprint format, which may not have undergone formal publication, we chose to present the comparison of algorithms and model performance with those featured on the TDC platform within the supplementary section. This evaluation framework allowed us to be as objective as possible in evaluating the performance of the proposed model. Also, for the sake of clarity, the model code is made available on GitHub, and the dataset is downloadable from the TDC platform so that all the analyses reported in this paper can be reproduced exactly. An additional consideration we would like to underscore is that the performances accomplished across diverse tasks were attained by employing a uniform network architecture for all layers, with the exception of the output layer. This strategic decision sets the stage for a promising avenue of future exploration: namely, the potential integration of multi-task network architectures. By harnessing such an approach, we could unlock enhanced capabilities by simultaneously addressing multiple ADMET prediction tasks, thereby pushing the boundaries of predictive accuracy and versatility in our model. However, it is essential to acknowledge the limitations of the proposed approach. Despite its advancements, the model may still encounter challenges in accurately predicting ADMET properties in scenarios with a limited availability of training data. The complexity of the developed model, comprising more than 750,000 parameters, underscores the necessity for a substantial volume of training data. In the real world, the pool of drug-like chemical compounds is inherently limited. Continual refinement and validation of the methodology against diverse datasets and experimental findings will be pivotal for overcoming these limitations and strengthening the reliability and applicability of this ADMET prediction framework. Furthermore, while we have conducted testing on publicly available datasets, it is crucial to consider broader validation efforts encompassing a wider spectrum of drug-like molecules. This could involve tapping into internal company datasets, which would offer valuable insights into the practical utility of our methodology in real-world settings.

## 5. Conclusions

In conclusion, the accurate prediction of ADMET properties is fundamental in the field of drug discovery and development. These properties play a pivotal role in understanding the pharmacokinetics, safety and efficacy of potential drug candidates, thereby enhancing the probability of achieving successful outcomes. The early and precise characterization of ADMET properties is essential to streamline a reliable and cost-effective drug discovery process and allows informed decision-making and fosters resource optimization. In this work, we introduce an innovative approach to ADMET prediction by leveraging the power of attention-based graph neural networks. The proposed model offers a novel methodology that combines the strengths of graph-based molecular representation and sophisticated neural network architectures. Central to this approach is the utilization of a graph-based representation of molecules derived directly from SMILE (Simplified Molecular Input Line Entry) notation. This step captures the intricate structural information of molecules coherently, facilitating the subsequent processing stages. The model employs an attention-based sequential information processing strategy, wherein it systematically analyzes substructures before aggregating them into a holistic representation of the entire molecule. As demonstrated with the ablation study, this approach can improve the prediction of ADMET properties, resulting also in a more biologically relevant prediction paradigm. Overall, our work offers a promising avenue for enhancing ADMET prediction accuracy and emphasizes the importance of leveraging innovative computational approaches to drive advancements in drug discovery and development. Moving forward, continuous refinement and validation of our methodology against diverse datasets and experimental data will be crucial for further enhancing the reliability and applicability of ADMET prediction frameworks.

## Figures and Tables

**Table 1:** List of the features used for characterizing each atom in the molecule.

Atomic Feature	List of Possible Values
Atom type identified by the atomic number	1–101
Formal charge	−3, −2, −1, 0, 1, 2, 3, Extreme
Hybridization Type	S, SP, SP2, SP3, SP3D, SP3D2, Other
Atom in a ring	0: No, 1: Yes
Is in an aromatic ring	0: No, 1: Yes
Chirality	Unspecified, Clockwise, Counter-clockwise, Other

**Table 2:** Statistics of the ADMET properties datasets. Compounds exhibiting an inhibitory action on CYP enzymes were labeled with 1 (i.e., positive examples). Conversely, 0 encodes an absence of inhibition (i.e., negative examples).

Property	#Total	#Positives (1)	#Negatives (0)	Task Type
Lipophilicity AZ	4200	-	-	Regression
AqSolDB	9982	-	-	Regression
CYP2C9	12,092	33.45%	66.54%	Binary Classification
CYP2C19	12,665	45.94%	54.06%	Binary Classification
CYP2D6	13,130	19.15%	80.85%	Binary Classification
CYP3A4	12,328	41.45%	58.55%	Binary Classification

## Data Availability

The data presented in this study are available in this article and Supplementary Material.

## Supplementary Materials

The following supporting information can be downloaded at: https://www.mdpi.com/article/10.3390/pharmaceutics16060776/s1. References [27,30,68,69,70] are cited in the Supplementary Materials.

## Appendix A

**Table A1:** Model’s performances on the lipophilicity dataset. Details of the 5-fold cross validation.

Fold	RMSE	MAE	R2
1	0.557	0.391	0.782
2	0.558	0.404	0.791
3	0.608	0.436	0.733
4	0.626	0.433	0.732
5	0.577	0.422	0.774
**Mean**	0.585	0.417	0.762
**Median**	0.576	0.422	0.774
**SD**	0.031	0.019	0.028

**Table A2:** Model’s performances on the AqSolDB dataset. Details of the 5-fold cross validation.

Fold	RMSE	MAE	R2
1	1.097	0.725	0.790
2	1.140	0.749	0.767
3	1.169	0.770	0.750
4	1.116	0.721	0.780
5	1.225	0.751	0.732
**Mean**	1.149	0.743	0.764
**Median**	1.140	0.749	0.767
**SD**	0.050	0.020	0.023

**Table A3:** Model’s performances on the CYP P450 2C9 dataset. Details of the 5-fold cross validation.

Fold	AUPRC	AUROC
1	0.799	0.895
2	0.797	0.894
3	0.790	0.894
4	0.772	0.870
5	0.787	0.886
**Mean**	0.789	0.888
**Median**	0.790	0.894
**SD**	0.010	0.009

**Table A4:** Model’s performances on the CYP P450 2C19 dataset. Details of the 5-fold cross validation.

Fold	AUPRC	AUROC
1	0.859	0.882
2	0.863	0.891
3	0.855	0.879
4	0.866	0.891
5	0.846	0.882
**Mean**	0.858	0.885
**Median**	0.859	0.882
**SD**	0.008	0.006

**Table A5:** Model’s performances on the CYP P450 2D6 dataset. Details of the 5-fold cross validation.

Fold	AUPRC	AUROC
1	0.708	0.871
2	0.674	0.865
3	0.676	0.850
4	0.686	0.862
5	0.676	0.858
**Mean**	0.684	0.861
**Median**	0.676	0.862
**SD**	0.014	0.008

**Table A6:** Model’s performances on the CYP P450 3A4 dataset. Details of the 5-fold cross validation.

Fold	AUPRC	AUROC
1	0.849	0.889
2	0.840	0.881
3	0.831	0.886
4	0.842	0.880
5	0.869	0.907
**Mean**	0.846	0.889
**Median**	0.842	0.886
**SD**	0.014	0.011

## References

[1] Wouters O.J., McKee M., Luyten J. (2020). Estimated Research and Development Investment Needed to Bring a New Medicine to Market, 2009–2018. JAMA.

[2] Cook D., Brown D., Alexander R., March R., Morgan P., Satterthwaite G., Pangalos M.N. (2014). Lessons learned from the fate of AstraZeneca’s drug pipeline: A five-dimensional framework. Nat. Rev. Drug Discov..

[3] Mohamed M.E., Trueman S., Othman A.A., Han J.H., Ju T.R., Marroum P. (2019). Development of In Vitro–In Vivo Correlation for Upadacitinib Extended-Release Tablet Formulation. AAPS J..

[4] Hanif M., Shoaib M.H., Yousuf R.I., Zafar F. (2018). Development of in vitro-in vivo correlations for newly optimized Nimesulide formulations. PLoS ONE.

[5] Kapungu N.N., Li X., Nhachi C., Masimirembwa C., Thelingwani R.S. (2020). In vitro and in vivo human metabolism and pharmacokinetics of S- and R-praziquantel. Pharmacol. Res. Perspect..

[6] Cheng F., Li W., Liu G., Tang Y. (2013). In silico ADMET prediction: Recent advances, current challenges and future trends. Curr. Top. Med. Chem..

[7] Patel C.N., Kumar S.P., Rawal R.M., Patel D.P., Gonzalez F.J., Pandya H.A. (2020). A multiparametric organ toxicity predictor for drug discovery. Toxicol. Mech. Methods.

[8] Berthelsen R., Sjögren E., Jacobsen J., Kristensen J., Holm R., Abrahamsson B., Müllertz A. (2014). Combining in vitro and in silico methods for better prediction of surfactant effects on the absorption of poorly water soluble drugs-a fenofibrate case example. Int. J. Pharm..

[9] Johansson S., Löfberg B., Aunes M., Lunde H., Frison L., Edvardsson N., Cullberg M. (2016). In Silico Predictions and In Vivo Results of Drug-Drug Interactions by Ketoconazole and Verapamil on AZD1305, a Combined Ion Channel Blocker and a Sensitive CYP3A4 Substrate. Clin. Pharmacol. Drug Dev..

[10] Litou C., Patel N., Turner D.B., Kostewicz E., Kuentz M., Box K.J., Dressman J. (2019). Combining biorelevant in vitro and in silico tools to simulate and better understand the in vivo performance of a nano-sized formulation of aprepitant in the fasted and fed states. Eur. J. Pharm. Sci..

[11] Wu F., Zhou Y., Li L., Shen X., Chen G., Wang X., Liang X., Tan M., Huang Z. (2020). Computational Approaches in Preclinical Studies on Drug Discovery and Development. Front. Chem..

[12] Muratov E.N., Bajorath J., Sheridan R.P., Tetko I.V., Filimonov D., Poroikov V., Oprea T.I., Baskin I.I., Varnek A., Roitberg A. (2020). QSAR without borders. Chem. Soc. Rev..

[13] Wei M., Zhang X., Pan X., Wang B., Ji C., Qi Y., Zhang J.Z. (2022). HobPre: Accurate prediction of human oral bioavailability for small molecules. J. Cheminform..

[14] Hou T., Wang J., Li Y. (2007). ADME Evaluation in Drug Discovery. 8. The Prediction of Human Intestinal Absorption by a Support Vector Machine. J. Chem. Inf. Model..

[15] Guerra A., Paez J., Campillo N.E. (2008). Artificial Neural Networks in ADMET Modeling: Prediction of Blood–Brain Barrier Permeation. J. Mol. Inform..

[16] Maria T.E., Roberta B., Paolo M. (2021). Application of Artificial Neural Networks to Predict the Intrinsic Solubility of Drug-Like Molecules. Pharmaceutics.

[17] Schyman P., Liu R., Desai V., Wallqvist A. (2017). vNN Web Server for ADMET Predictions. Front. Pharmacol..

[18] Gómez-Bombarelli R., Wei J.N., Duvenaud D., Hernández-Lobato J.M., Sánchez-Lengeling B., Sheberla D., Aguilera-Iparraguirre J., Hirzel T.D., Adams R.P., Aspuru-Guzik A. (2018). Automatic Chemical Design Using a Data-Driven Continuous Representation of Molecules. ACS Cent. Sci..

[19] Salma H., Melha Y.M., Sonia L., Hamza H., Salim N. (2021). Efficient Prediction of In Vitro Piroxicam Release and Diffusion From Topical Films Based on Biopolymers Using Deep Learning Models and Generative Adversarial Networks. J. Pharm. Sci..

[20] Jiménez-Luna J., Grisoni F., Schneider G. (2021). Drug discovery with explainable artificial intelligence. Nat. Mach. Intell..

[21] Guha R., Willighagen E. (2012). A Survey of Quantitative Descriptions of Molecular Structure. Curr. Top. Med. Chem..

[22] Khan M.T. (2010). Predictions of the ADMET properties of candidate drug molecules utilizing different QSAR/QSPR modelling approaches. Curr. Drug Metab..

[23] Duan J., Dixon S.L., Lowrie J.F., Sherman W. (2010). Analysis and comparison of 2D fingerprints: Insights into database screening performance using eight fingerprint methods. J. Mol. Graph Model..

[24] Aouichaoui A.R., Fan F., Abildskov J., Sin G. (2023). Application of interpretable group-embedded graph neural networks for pure compound properties. Comput. Chem. Eng..

[25] Fralish Z., Chen A., Skaluba P., Reker D. (2023). DeepDelta: Predicting ADMET improvements of molecular derivatives with deep learning. J. Cheminform..

[26] Peng Y., Lin Y., Jing X.-Y., Zhang H., Huang Y., Luo G.S. (2020). Enhanced Graph Isomorphism Network for Molecular ADMET Properties Prediction. IEEE Access.

[27] Weininger D. (1988). SMILES, a chemical language and information system. 1. Introduction to methodology and encoding rules. J. Chem. Inf. Comput. Sci..

[28] Huang K., Fu T., Gao W., Zhao Y., Roohani Y., Leskovec J., Coley C.W., Xiao C., Sun J., Zitnik M. (2022). Artificial intelligence foundation for therapeutic science. Nat. Chem. Biol..

[29] David L., Thakkar A., Mercado R., Engkvist O. (2020). Molecular representations in AI-driven drug discovery: A review and practical guide. J. Cheminform..

[30] Hamilton W.L. (2020). Graph Representation Learning.

[31] Zhou J., Cui G., Hu S., Zhang Z., Yang C., Liu Z., Wang L., Li C., Sun M. (2020). Graph neural networks: A review of methods and applications. AI Open.

[32] Wu Z., Pan S., Chen F., Long G., Zhang C., Yu P.S. (2019). A Comprehensive Survey on Graph Neural Networks. IEEE Trans. Neural Netw. Learn. Syst..

[33] Ioffe S., Szegedy C. Batch normalization: Accelerating deep network training by reducing internal covariate shift. Proceedings of the 32nd International Conference on International Conference on Machine Learning.

[34] Haykin S. (1994). Neural Networks: A Comprehensive Foundation.

[35] Bahdanau D., Cho K., Bengio Y. (2014). Neural machine translation by jointly learning to align and translate. arXiv.

[36] Vaswani A., Shazeer N., Parmar N., Uszkoreit J., Jones L., Gomez A.N., Kaiser L., Polosukhin I. Attention is all you need. Proceedings of the Advances in Neural Information Processing Systems.

[37] Brauwers G., Frasincar F. (2023). A General Survey on Attention Mechanisms in Deep Learning. Inst. Electr. Electron. Eng..

[38] Veličković P., Cucurull G., Casanova A., Romero A., Liò P., Bengio Y. (2017). Graph attention networks. arXiv.

[39] Mass A.L., Hannun A.Y., Ng A.Y. Rectifier nonlinearities improve neural network acoustic models. Proceedings of the 30th International Conference on Machine Learning.

[40] Li Y., Tarlow D., Brockschmidt M., Zemel R. (2017). Gated Graph Sequence Neural Networks. arXiv.

[41] Kawabata Y., Wada K., Nakatani M., Yamada S., Onoue S. (2011). Formulation design for poorly water-soluble drugs based on biopharmaceutics classification system: Basic approaches and practical applications. Int. J. Pharm..

[42] Sim S.C., Ingelman-Sundberg M. (2010). The Human Cytochrome P450 (CYP) Allele Nomenclature website: A peer-reviewed database of CYP variants and their associated effects. Hum. Genom..

[43] Bengio Y., Montavon G., Orr G.B., Müller K.R. (2007). Practical Recommendations for Gradient-Based Training of Deep Architectures. Neural Networks: Tricks of the Trade.

[44] Cui Y., Jia M., Lin T.-Y., Song Y., Belongie S. Class-balanced loss based on effective number of samples. Proceedings of the IEEE/CVF Conference on Computer Vision and Pattern Recognition.

[45] Krawczyk B. (2018). Learning from imbalanced data: Open challenges and future directions. Prog. Artif. Intell..

[46] Steininger M., Kobs K., Davidson P., Krause A., Hotho A. (2021). Density-based weighting for imbalanced regression. Mach. Learn..

[47] Chen Y.-C. (2017). A tutorial on kernel density estimation and recent advances. Biostat. Epidemiol..

[48] Silverman B.W. (1986). Density Estimation for Statistics and Data Analysis.

[49] Japkowicz N., Stephen S. (2002). The Class Imbalance Problem: A Systematic Study. Intell. Data Anal..

[50] Zhou Z.-H., Liu X.-Y. (2006). Training cost-sensitive neural networks with methods addressing the class imbalance problem. Knowl. Data Eng. IEEE Trans..

[51] Fern K.R., Tsokos C.P. (2022). Dynamically Weighted Balanced Loss: Class Imbalanced Learning and Confidence Calibration of Deep Neural Networks. IEEE Trans. Neural Netw. Learn. Syst..

[52] Goadrich M., Oliphant L., Shavlik J. (2006). Gleaner: Creating ensembles of first-order clauses to improve recall-precision curves. Mach. Learn..

[53] Boyd K., Eng K.H., Page C.D. (2013). Area under the precision-recall curve: Point estimates and confidence intervals. Proceedings of the 2013th European Conference on Machine Learning and Knowledge Discovery in Databases.

[54] Davis J., Goadrich M. The relationshipt between Precision-Recall and ROC curves. Proceedings of the 23rd International Conference on Machine Learning.

[55] Saito T., Rehmsmeier M. (2015). The precision-recall plot is more informative than the ROC plot when evaluating binary classifiers on imbalanced datasets. PLoS ONE.

[56] Zhang X., Wu C., Yang Z., Wu Z., Yi J., Hsieh C., Hou T., Cao D. (2021). MG-BERT: Leveraging unsupervised atomic representation learning for molecular property prediction. Briefings Bioinform..

[57] Wang X., Liu M., Zhang L., Wang Y., Li Y., Lu T. (2020). Optimizing Pharmacokinetic Property Prediction Based on Integrated Datasets and a Deep Learning Approach. J. Chem. Inf. Model..

[58] Xu K., Hu W., Leskovec J., Jegelka S. (2018). How Powerful are Graph Neural Networks?. arXiv.

[59] Tang B., Kramer S.T., Fang M., Qiu Y., Wu Z., Xu D. (2020). A self-attention based message passing neural network for predicting molecular lipophilicity and aqueous solubility. J. Cheminform..

[60] Li X., Fourches D. (2020). Inductive transfer learning for molecular activity prediction: Next-Gen QSAR Models with MolPMoFiT. J. Cheminform..

[61] Xiong Z., Wang D., Liu X., Zhong F., Wan X., Li X., Li Z., Luo X., Chen K., Jiang H. (2020). Pushing the Boundaries of Molecular Representation for Drug Discovery with the Graph Attention Mechanism. J. Med. Chem..

[62] Francoeur P.G., Koes D.R. (2021). SolTranNet—A Machine Learning Tool for Fast Aqueous Solubility Prediction. J. Chem. Inf. Model..

[63] Maziarka L., Danel T., Mucha S., Rataj K., Tabor J., Jastrzebski S. (2002). Molecule Attention Transformer. arXiv.

[64] Yang K., Swanson K., Jin W., Coley C., Eiden P., Gao H., Guzman-Perez A., Hopper T., Kelley B., Mathea M. (2019). Analyzing learned molecular representations for property prediction. J. Chem. Inf. Model..

[65] Venkatraman V. (2021). FP-ADMET: A compendium of fingerprint-based ADMET prediction models. J. Cheminform..

[66] Plonka W., Stork C., Šícho M., Kirchmair J. (2021). CYPlebrity: Machine learning models for the prediction of inhibitors of cytochrome P450 enzymes. Bioorganic Med. Chem..

[67] Li X., Xu Y., Lai L., Pei J. (2018). Prediction of Human Cytochrome P450 Inhibition Using a Multitask Deep Autoencoder Neural Network. Mol. Pharm..

[68] TDC Leaderboard Guidelines. https://tdcommons.ai/benchmark/overview.

[69] TDC ADMET Benchmark Groups. https://tdcommons.ai/benchmark/admet_group/overview/.

[70] Landrum G., Tosco P., Kelley B., Sriniker, Gedeck, Schneider N., Vianello R., Ric, Dalke A., Cole B. (2020). rdkit/rdkit: 2020 03 1. Q1 2020 Release. https://zenodo.org/records/3732262.

