# Supplementary material for: Predicting ADMET Properties from Molecule SMILE: A Bottom-Up Approach Using Attention-Based Graph Neural Networks
Source: Pharmaceutics. 2024 Jun 7;16(6):776. doi: 10.3390/pharmaceutics16060776 (PMC11207804; doi:10.3390/pharmaceutics16060776)
Supplement: Supplementary file 1 [file pharmaceutics-16-00776-s001.zip › pharmaceutics-2823297-supplementary.pdf]

Supplementary Material S1

Evaluation of the proposed GNN architecture following the standard of TDC platform

The results obtained with the architecture here presented are reported in Table S1.1-S1.6 and compared with the TDC leaderboard in terms of ranking and gap from leader/previous model (Table S1.7). A detailed description of the evaluation procedure proposed by the platform is reported in [68].

This study introduces a novel architecture that employs a bottom-up approach for estimating ADMET properties, focusing on a broader range of tasks rather than a single task. Our model demonstrates competitive performance, comparable to top-ranking approaches. While itl may appear to have relatively lower rankings in certain tasks, it is worth noting that the differences with the top-performing algorithms [69] are minimal.

Table S1.1: Results on the Lipophilicity task

Lipophilicity	RMSE	MAE	R2
**Split #1**	0.644	0.475	0.705
**Split #2**	0.621	0.464	0.726
**Split #3**	0.594	0.44	0.749
**Split #4**	0.577	0.432	0.764
**Split #5**	0.615	0.47	0.731
**Median**	0.615	0.464	0.731
**Mean**	0.6102	0.4562	0.735
**SD**	0.025724	0.019058	0.02255

Table S1.2: Results on the AqSolDB task

AqSolDB	RMSE	MAE	R2
**Split #1**	1.398	0.879	0.63
**Split #2**	1.329	0.89	0.664
**Split #3**	1.598	0.866	0.516
**Split #4**	1.204	0.86	0.725
**Split #5**	1.293	0.87	0.683
**Median**	1.329	0.87	0.664
**Mean**	1.3644	0.873	0.6436

**SD**	0.148136	0.011747	0.079154

Table S1.3: Results on the CYP-2C9 task

**CYP-2C9**	**AUPRC**	**AUROC**
**Split #1**	0.761	0.881
**Split #2**	0.753	0.877
**Split #3**	0.746	0.882
**Split #4**	0.728	0.852
**Split #5**	0.762	0.883
**Median**	0.753	0.881
**Mean**	0.75	0.875
**SD**	0.01391	0.013058

Table S1.4: Results on the CYP-2C19 task

**CYP-2C19**	**AUPRC**	**AUROC**
**Split #1**	0.744	0.866
**Split #2**	0.767	0.89
**Split #3**	0.747	0.879
**Split #4**	0.736	0.868
**Split #5**	0.751	0.878
**Median**	0.747	0.878

**Mean**	0.749	0.8762
**SD**	0.011467	0.009654

Table S1.5: Results on the CYP-2D6 task

**CYP-2D6**	**AUPRC**	**AUROC**
**Split #1**	0.653	0.859
**Split #2**	0.652	0.869
**Split #3**	0.622	0.861
**Split #4**	0.656	0.87
**Split #5**	0.606	0.831
**Median**	0.652	0.861
**Mean**	0.6378	0.858
**SD**	0.022499	0.015843

Table S1.6: Results on the CYP-3A4 task

**CYP-3A4**	**AUPRC**	**AUROC**
**Split #1**	0.84	0.868
**Split #2**	0.847	0.876
**Split #3**	0.845	0.879
**Split #4**	0.854	0.884
**Split #5**	0.847	0.879

**Median**	0.847	0.879
**Mean**	0.8466	0.8772
**SD**	0.00503	0.005891

Table S1.7: Comparison of the proposed approach with TDC benchmark models.

Dataset	TDC Ranking	Gap from leader/previous
Lipophilicity	1	-0.010 [MAE]
AqSolDB	6	+0.111 [MAE]
CYP2C9	6	-0.089 [AUPRC]
CYP2C19	1	- [AUPRC]
CYP2D6	7	-0.1012 [AUPRC]
CYP3A4	8	-0.0574 [AUPRC]

References

[68] TDC Leaderboard Guidelines. Online at https://tdcommons.ai/benchmark/overview
[69] TDC ADMET Benchmark Groups. Online at https://tdcommons.ai/benchmark/admet_group/overview/.

Supplementary Material S2

Implementative details of the proposed GNN

Data Preprocessing

In the considered datasets, each molecule is represented through its relative SMILES, that is a string data. Python package *rdkit* [27] was used to extract the atomic features from SMILES. Then, the adjacency matrices of both the whole molecule and its sub-structures were obtained converting SMILES to graph with *rdkit* and *networkx* [70] Python packages.

GNN hyperparameters

All GNN layers were implemented in Python using Tensorflow 2.4 library. Scikit-learn Python package supported the implementation of the 5-folds Cross Validation pipeline and the kernel density estimation of regression output. Table S2.1 contains the values of the hyperparameters for the GNN in each task.

Table S2.1 List of the hyperparameters that were adopted in the different tasks.

**Hyperparameter**	**Value**
**Hyperparameters common to all tasks**	
Output Dimension of Attention Head - Layer 1	32
Output Dimension of Attention Head - Layer 3	64
Output Dimension of Attention Head - Layer 6	128
Output Size of Global Attention Pooling Layer	512
Optimizer	Adam
Batch Size	16
**Hyperparameters for regression task on Lipophilicity dataset**	
Type of Kernel Density Estimator	Gaussian
Bandwidth of the KDE	0.2
α - weighting factor of WRMSE	0.55
Epochs	500
Learning Rate	0.001
MLP architecture	1 layer with 16 units
**Hyperparameters for regression task on AcqSol dataset**	
Type of Kernel Density Estimator	Gaussian
Bandwidth of the KDE	0.3
α - weighting factor of WRMSE	0.55
Epochs	750
Learning Rate	0.0001
MLP architecture	4 layers with 128,64,32 and 16 units respectively

Hyperparameters for all classification tasks on CYP datasets	
Epochs	500
Learning Rate	0.0001
MLP architecture	4 layers with 128,64,32 and 16 units respectively

References

[27]David Weininger. SMILES, a chemical language and information system. 1. Introduction to methodology and encoding rules. Journal of chemical information and computer sciences 1988 28(1) 31–36

[70]Greg Landrum,Paolo Tosco, Brian Kelley,sriniker, gedeck, Nadine Schneider, Riccardo Vianello,Ric,Andrew Dalke, Brian Cole, Alexander Savelyev, Matt Swain, Samo Turk, Dan N, Alain Vaucher, Eisuke Kawashima, Maciej Wójcikowski, Daniel Probst, Guillaume Godin, David Cosgrove, Axel Pahl, JP, Francois Berenger, strets123, JL Varjo, Noel O'Boyle, Patrick Fuller, Jan Holst, Jensen Gianluca Sforna, Doliath Gavid. rdkit/rdkit: 2020_03_1 (Q1 2020) Release. 2020. https://doi.org/10.5281/zenodo.3732262 544

Supplementary Material S3

Graph Convolutional Layer

Graph Convolutional Layer

Graph convolutional (GC) layer represents the most simple and common neural network substructure processing graph data [30]. The inputs of GC layers are graph adjacency matrix, $A \in R^{N \times N}$, and nodes features matrix, $H \in R^{N \times M}$, with N representing the number of

graph nodes and M the number of features for each node. For the sake of simplicity, let us assume that the elements on the diagonal of A are equal to 0 (i.e., each node is not considered linked to itself in the graph). Therefore, each node u is characterized by a set of features $h_u \in R^{1\times M}$ and by a set of neighbors, V_u, with $v \neq u \; \forall \; v \in V_u$. The GC layer projects each h_u to a D-dimensional latent space by merging the information (i.e., features) on its neighbors $v \in V_u$. To this end, for each u, the steps of aggregation and updates are performed. In particular, the aggregation step consists in merging the features of all $v \in V_u$. In the GC layer, the aggregation operation with respect to the node u is the sum of the features vectors of $v \in V_u$ (Eq. S3.1).

$$m_u = \sum_{v \in V_u} h_v$$

Eq. S3. 1

In the update step (Eq. S3.2) a new features vector, $h'_u \in R^{1\times D}$, is obtained for each u. In particular, the new D-dimensional representation of u is based on a linear combination between the old representation of the node (h_u) and the information of its neighbors (m_u). These two terms are weighted by the coefficients matrices $W_1 \in R^{M\times D}$ and $W_2 \in R^{M\times D}$ which represent the learnable parameters of a GC layer. A bias vector $b \in R^{1\times D}$ is added to the result of the linear combination and, finally, a nonlinear function, g , (i.e., the activation function of the GC layer) is applied to each feature.

$$h'_u = g(h_u \cdot W_1 + m_u \cdot W_2 + b).$$

Eq. S3. 2

Eq. S3.1-2 can be rewritten in a more compact form by using the tensorial form:

$$H' = g(H \cdot W_1 + A \cdot H \cdot W_2 + b).$$

Eq. S3. 3

In particular, a common design choice to reduce the number of trainable parameters is to consider $W_1 = W_2$ [30]. Thus, Eq. S3.3 can be further simplified as reported in Eq. S3.4:

$$H' = g((A + I) \cdot H \cdot W_1 + b)$$

with $I \in R^{N\times N}$ being the identity matrix and $H' \in R^{N\times D}$ the matrix containing the D-dimensional features vectors for each of the N nodes.

References

[30] William L. Hamilton. Graph Representation Learning; Springer Cham, 2020.

Hyperparameters of the models implemented in the ablation study

Table S3.1 List of the hyperparameters that were adopted in the different tasks for the Whole Molecule GNN.

**Hyperparameter**	**Value**
**Hyperparameters common to all tasks**	
Output Dimension of Attention Head - Layer 1	32
Output Dimension of Attention Head - Layer 3	64
Output Dimension of Attention Head - Layer 5	128
Output Size of Global Attention Pooling Layer	512
Optimizer	Adam
Batch Size	16
**Hyperparameters for regression task on Lipophilicity dataset**	
Type of Kernel Density Estimator	Gaussian
Bandwidth of the KDE	0.2
α - weighting factor of WRMSE	0.55
Epochs	500
Learning Rate	0.001
MLP architecture	1 layer with 16 units
**Hyperparameters for regression task on AcqSol dataset**	
Type of Kernel Density Estimator	Gaussian
Bandwidth of the KDE	0.3
α - weighting factor of WRMSE	0.55
Epochs	750
Learning Rate	0.0001
MLP architecture	4 layers with 128,64,32 and 16 units respectively
**Hyperparameters for all classification tasks on CYP datasets**	

Epochs	500
Learning Rate	0.0001
MLP architecture	4 layers with 128,64,32 and 16 units respectively

Table S3.2 List of the hyperparameters that were adopted in the different tasks for the Convolutional GNN.

**Hyperparameter**	**Value**
**Hyperparameters common to all tasks**	
Output Dimension of Attention Head - Layer 1	32
Output Dimension of Attention Head - Layer 3	64
Output Dimension of Graph Convolutional Layer - Layer 6	512
Output Size of Global Attention Pooling Layer	512
Optimizer	Adam
Batch Size	16
**Hyperparameters for regression task on Lipophilicity dataset**	
Type of Kernel Density Estimator	Gaussian
Bandwidth of the KDE	0.2
α - weighting factor of WRMSE	0.55
Epochs	500
Learning Rate	0.001
MLP architecture	1 layer with 16 units
**Hyperparameters for regression task on AcqSol dataset**	
Type of Kernel Density Estimator	Gaussian
Bandwidth of the KDE	0.3
α - weighting factor of WRMSE	0.55
Epochs	750
Learning Rate	0.0001
MLP architecture	4 layers with 128,64,32 and 16 units respectively

**Hyperparameters for all classification tasks on CYP datasets**	
Epochs	500
Learning Rate	0.0001
MLP architecture	4 layers with 128,64,32 and 16 units respectively
